# Supplementary material for: Maternal High-Fiber Diet Protects Offspring against Type 2 Diabetes
Source: Nutrients. 2020 Dec 30;13(1):94. doi: 10.3390/nu13010094 (PMC7823372; doi:10.3390/nu13010094)
Supplement: Supplementary file 1 [file nutrients-13-00094-s001.zip › Supplementary_Information/Supplementary_Data.docx]

# Maternal High Fiber Diet Protects Offspring Against Type 2 Diabetes

Huishi Toh^1,*^, James A. Thomson^2,3,4^, Peng Jiang^4,*^

^1^ Neuroscience Research Institute, University of California Santa Barbara, Santa Barbara, CA, USA

^2^ Department of Molecular, Cellular and Developmental Biology, University of California Santa Barbara, Santa Barbara, CA, USA

^3^ Department of Cell and Regenerative Biology, University of Wisconsin School of Medicine and Public Health, Madison, WI, USA

^4^ Regenerative Biology Laboratory, Morgridge Institute for Research, Madison, WI, USA

**^*^To whom correspondence should be addressed**

Huishi Toh ([toh@ucsb.edu](mailto:toh@ucsb.edu))

Peng Jiang ([PJiang@morgridge.org](mailto:PJiang@morgridge.org))

# List of Supplementary Tables

Table S1: Detailed information of Nile rats used in this study including sex, maternal diet, offspring diet and random blood glucose (RBG) levels from 4-40 weeks old. All animals with RBG>500mg/dL were euthanized for humane reasons. We then transformed all RBG values over 500 mg/dL to 500 mg/dL.

# List of Supplementary Figures

Figure S1: Graphic illustration of the number of Nile rats for each category.

Figure S2: A simple Bayesian network for modeling relative diabetic risk. The nodes structure are pre-defined.

Figure S3: A high fiber offspring’s diet can prevent to develop hyperglycemia regardless of maternal diet. (A) The offspring are males. N=76 (green), N=55 (red); (b) The offspring are females. The number of Nile rats: N=55 (green), N= 35(red).

Figure S4: The weight is heavier in offspring with maternal diabetogenic diet than with maternal high fiber diet at the time of weaning. The number of Nile rats (N) is shown for each maternal diet category.
