# Supplementary material for: Maternal High-Fiber Diet Protects Offspring against Type 2 Diabetes
Source: Nutrients. 2020 Dec 30;13(1):94. doi: 10.3390/nu13010094 (PMC7823372; doi:10.3390/nu13010094)
Supplement: Supplementary file 1 [file nutrients-13-00094-s001.zip › Supplementary_Information/Supplementary_Fig.S2_BN_Methods.pdf]

Figure S2

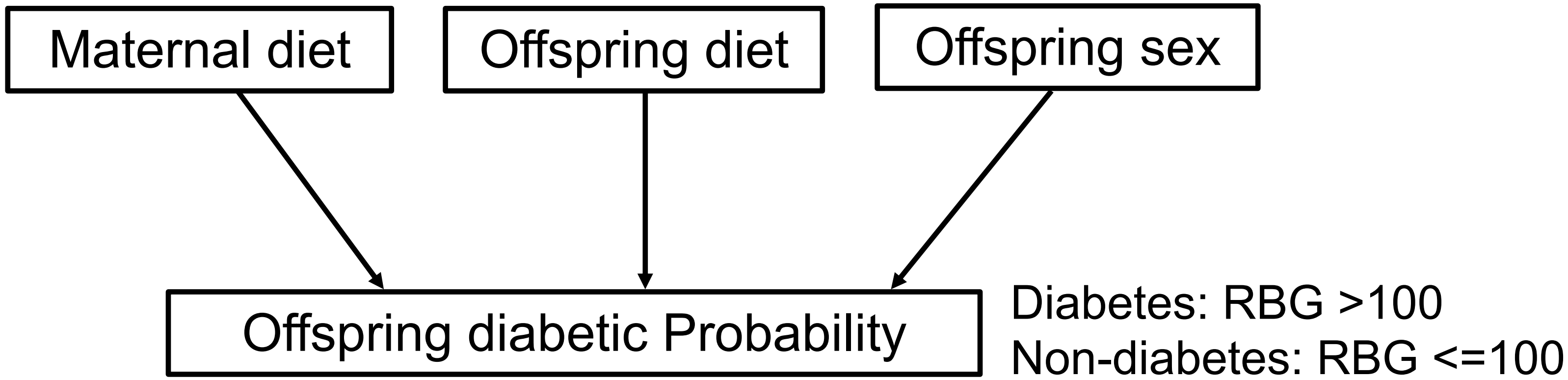

P (Risk | Maternal diet, Offspring diet, Offspring sex)

Network structure: Predefined
Bayesian network parameter learning and probability inference: R package (“bnlearn”)
