# Supplementary figures and images for: Maternal High-Fiber Diet Protects Offspring against Type 2 Diabetes
Source: Nutrients. 2020 Dec 30;13(1):94. doi: 10.3390/nu13010094 (PMC7823372; doi:10.3390/nu13010094)

Figure S1

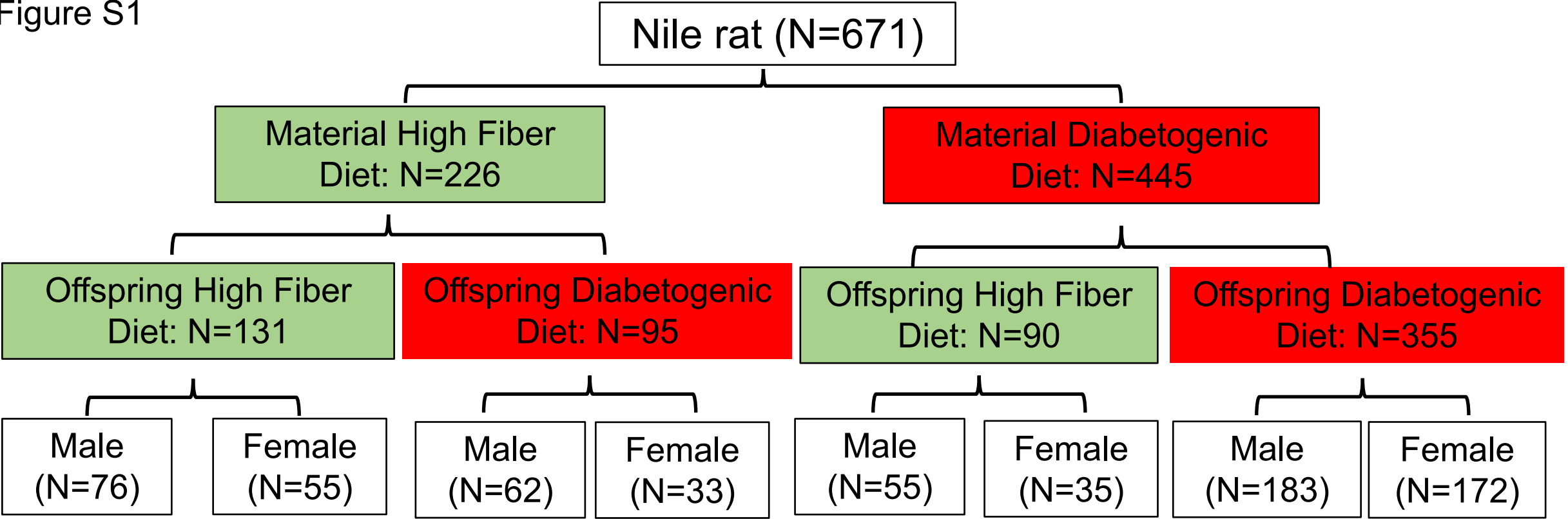

Supplement: Supplementary file 1 [file nutrients-13-00094-s001.zip › Supplementary_Information/Supplementary_Fig.S1_Nile_Rat_Number_Summary.pdf]

Figure S3

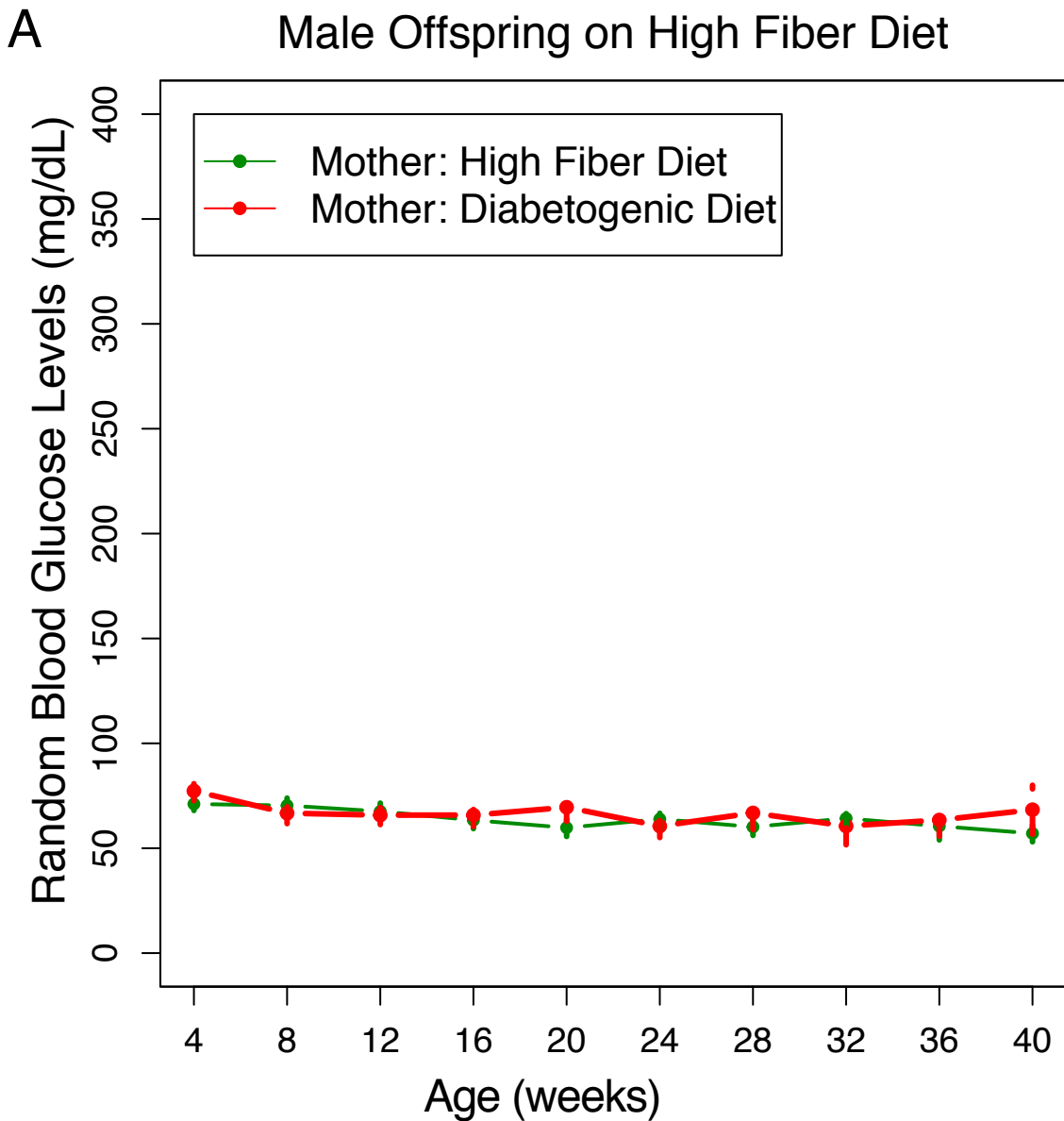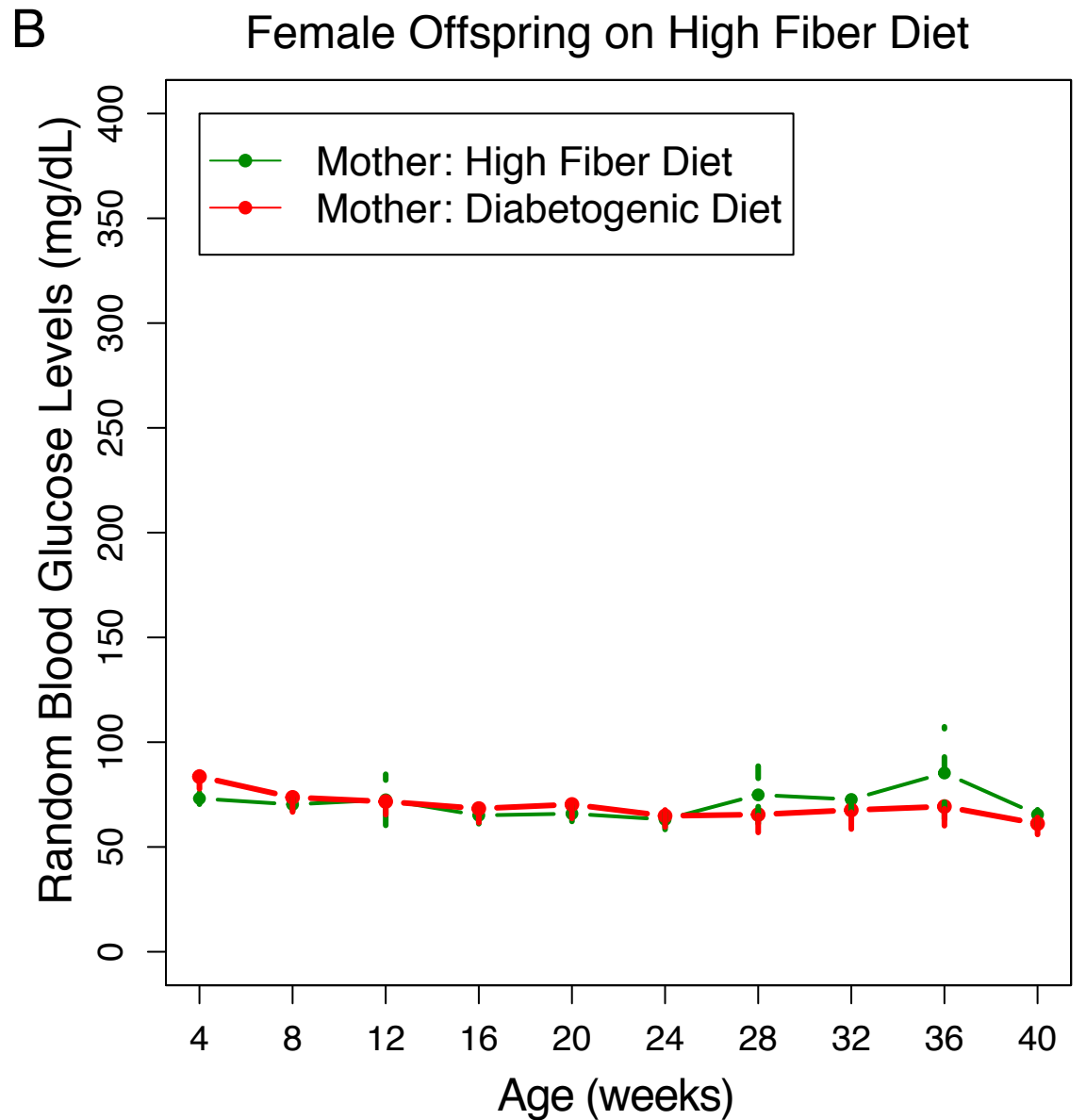

Supplement: Supplementary file 1 [file nutrients-13-00094-s001.zip › Supplementary_Information/Supplementary_Fig.S3.pdf]

## 4-Week Old

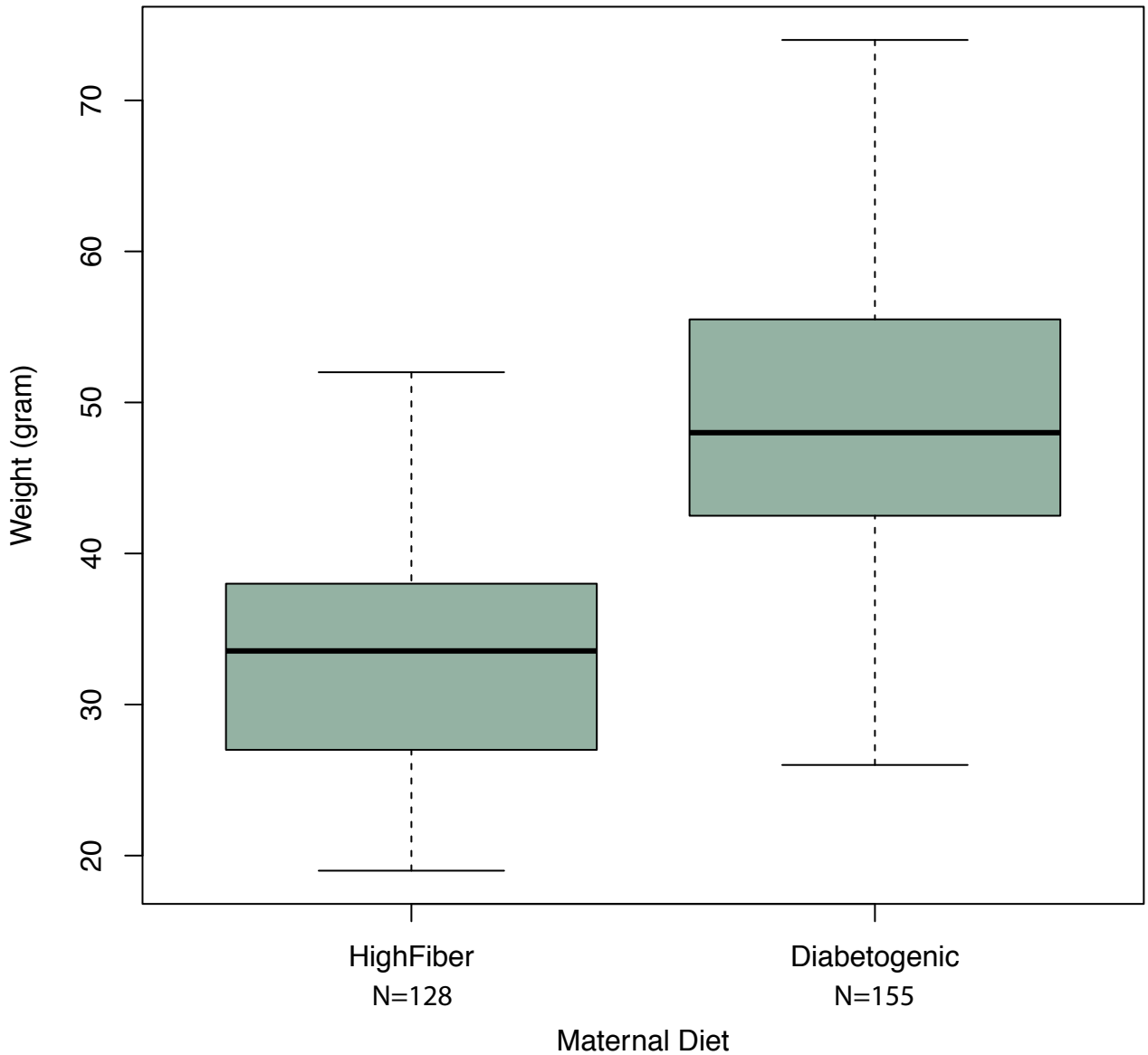

Supplement: Supplementary file 1 [file nutrients-13-00094-s001.zip › Supplementary_Information/Supplementary_Fig_S4.pdf]
